# The Protective Effects of Eicosapentaenoic Acid for Stress-induced Accelerated Senescence in Vascular Endothelial Cells

**DOI:** 10.7150/ijms.85224

**Published:** 2023-09-11

**Authors:** Min Jeong Kwon, Hye Sook Jung, Seon Mee Kang, Soon Hee Lee, Jeong Hyun Park

**Affiliations:** 1 Department of Internal Medicine, Busan Paik Hospital, College of Medicine, Inje University, Busan, South Korea.; 2 Paik Institute for Clinical Research, Inje University, Busan, South Korea.; 3 Department of Internal Medicine, Kangwon National University Hospital, School of Medicine, Kangwon National University, Chuncheon, South Korea.

**Keywords:** eicosapentaenoic acid, oxidative stress, stress-induced accelerated senescence

## Abstract

**Background:** Eicosapentaenoic acid (EPA) is an omega-3 fatty acid that protects against cardiovascular diseases in patients with hypertriglyceridemia and may have pleotropic effects beyond lowering triglycerides. Many degenerative diseases, such as atherosclerosis and diabetes, are related to cellular senescence as a pathophysiological mechanism. We aimed to examine whether EPA could protect vascular endothelial cells under stress conditions against stress-induced accelerated senescence (SIAS).

**Methods:** Cultured human umbilical vein endothelial cells (HUVECs) were exposed to H_2_O_2_ as oxidative stress and a high glucose concentration with palmitate as a glucolipotoxic condition. Changes in cell viability, apoptosis, lactate dehydrogenase release, and cell cycle analysis were measured by cell counting kit-8 assay, annexin V/ propidium iodide staining, and enzyme-linked immunosorbent assay, respectively. EPA was applied in stress conditions. The degree of senescence was measured by senescence-associated beta-galactosidase staining and p16 staining using immunofluorescence. Apoptosis and cellular senescence-related proteins were measured by Western blotting.

**Results:** Cultured HUVECs under oxidative and glucolipotoxic stresses revealed accelerated senescence and increased apoptosis. These changes were markedly reversed by EPA administration, and the expressions of apoptosis and cellular senescence-related proteins were reversed by EPA treatment.

**Conclusion:** EPA effectively protects HUVECs against SIAS, which may be one of its pleotrophic effects.

## Introduction

Atherosclerosis is a progressive chronic inflammatory disease that causes major cardiovascular diseases such as ischemic heart disease, peripheral vascular disease, and cerebral infarction and is closely related to aging 1. Diabetes mellitus also causes atherosclerosis, and cardiovascular disease is a main cause of death in patients with diabetes. Diabetes is evaluated as a coronary artery disease risk equivalent, which means an equivalent risk level as already having cardiovascular disease in terms of the risk of developing cardiovascular disease. Cellular senescence is a process in which various stressors permanently stop cell division, resulting in cell changes that appear as senescent-associated secretory phenotype (SASP) 2. SASP is involved in the secretion of substances such as growth factors, cytokines, and chemokines and the generation of reactive oxygen species (ROS) 3. Diabetes prevalence increases with age; growth factors, cytokines, and ROS are some of the mechanisms of diabetic complications **4**. Glucotoxicity and lipotoxicity are also potential mechanisms for diabetic complications, in addition to beta cell dysfunction. Hyperglycemia and hyperlipidemia, common in patients with uncontrolled diabetes, cause oxidative and endoplasmic reticulum stress, causing complications 5.

The beneficial effect of omega-3 fatty acids on atherosclerosis has been suggested to involve several mechanisms such as to induce anti-inflammatory and anti-arrhythmic effects, to improve the lipid profile by lowering triglyceride levels, to prevent plaque development, to promote plaque stabilization, and to inhibit platelet aggregation 6. Additionally, they may have the benefit of improving endothelial cell dysfunction caused by blunted endothelium-dependent vasodilatation mostly due to a reduced bioavailability of endothelium-derived nitric oxide (NO), and, often, endothelium-derived hyperpolarization in an early step of atherogenesis. The eicosapentaenoic acid (EPA) has been shown to cause endothelium-dependent relaxations of isolated sheep pulmonary arteries, and to increase endothelial NO synthase (eNOS) activity and NO formation in cultured endothelial cells 7-9. In addition, EPA but not docosahexaenoic acid (DHA) has also been shown to cause cerebral microvascular vasodilatation involving arachidonic acid metabolites 10. A study showed that highly purified EPA:DHA 6:1 and 9:1 formulations demonstrated greater endothelium-dependent NO-mediated relaxation of porcine coronary artery rings than other ratios 11.

Several clinical studies, but not all, have suggested that omega-3 polyunsaturated fatty acids, in particular EPA and DHA, improve the endothelial function and reduce cardiovascular events in patients with cardiovascular risk factors 12-14. In a recently reported study, Reduction of Cardiovascular Events with Icosapent Ethyl-Intervention Trial (REDUCE-IT), icosapent ethyl (IPE), which is a highly purified and stable eicosapentaenoic acid (EPA) ethyl ester, significantly lowered the risk of ischemic events, including cardiovascular death in patients with elevated triglyceride levels despite the use of statins 15. However, the observed reduction in low-density lipoprotein cholesterol (LDL-C), triglycerides, and non-high-density lipoprotein cholesterol (non-HDL-C) did not fully account for the positive outcomes, indicating that other properties of EPA were likely involved in the benefits 16.

Hence, we aimed to investigate the impact of oxidative stress or glucolipotoxicity at the cellular level on atherosclerosis in patients with diabetes in relation to SASP and explore whether EPA responds to SASP in these environment.

## Materials and Methods

### Reagents

EPA (E2011, 99% purity, ethyl ester) was purchased from Sigma (MO, USA) and dissolved in dimethyl sulfoxide.

### Cell culture

Human umbilical vein endothelial cells (HUVECs) were obtained from Promocell (Heidelberg, Germany). HUVECs were cultured in endothelial growth medium (EGM) with 5 ng/mL epidermal growth factor, 10 ng/mL basic fibroblast growth factor, 20 ng/mL insulin-like growth factor, 0.5 ng/mL vascular endothelial growth factor 165, 1 μg/mL ascorbic acid, 22.5 μg/mL heparin and 0.2 μg/mL hydrocortisone (Promocell, Heidelberg, Germany) in all experiments. All cells were incubated at 37°C with 5% CO_2_ and used between the third and seventh passage.

### Cell viability using cell counting kit (CCK)-8 assay

HUVECs were seeded in 96-well plates at 7×10^3^ cells per well and incubated for 24 hours in EGM. To confirm cell viability by H_2_O_2_ (Sigma, MO, USA) or high glucose concentration with palmitate (HGP) with/without EPA (Sigma, MO, USA), the culture medium was aspirated and replaced with fresh medium containing varying concentrations of EPA for 2 hours, followed by incubation with H_2_O_2_ (300 μM) or HGP (glucose 25 mM and palmitate 300 μM) for 48 hours. The medium was then replaced with CCK-8 reaction buffer (Dojindo, Munich, Germany), and the cells were incubated for 2 hours at 37°C. The optical density was measured at a wavelength of 450 nm using a microplate reader to determine cell viability.

### Cell apoptosis using annexin V/ propidium iodide (PI) staining and lactate dehydrogenase (LDH) release assay

HUVECs were seeded in 12-well plates at 7×10^4^ cells per well and incubated for 24 hours. To confirm the anti-apoptotic effect of H_2_O_2_ or HGP with/without EPA, culture mediums were aspirated and replaced with new mediums containing various concentrations of EPA for 2 hours and then incubated in 300 μM H_2_O_2_ or HGP (glucose 25 mM and palmitate 300 μM) for 48 hours. To obtain single cells, cells were treated with trypsin-EDTA 200 μL and centrifuged at 1500 rpm for 5 minutes at 4°C. After supernatant aspiration, cells were washed with 1 mL of annexin V binding buffer (140 mM NaCl, 10 mM HEPES, pH 7.4, 2.5 mM CaCl_2_) and centrifuged at 1,500 rpm for 5 minutes at 4°C. Supernatants were removed, and 3 μL of annexin V-FITC (BD, CA, USA) and 10 μL of PI (BD, CA, USA) was added. After incubation for 15 minutes in the dark, 300 μL of fluorescence activated cell sorting (FACS) buffer (1% FBS, 0.1% NaN_3_) was added, and analysis was performed using FACSort (BECTON DICKINSON, BD bioscience). After treatment with various concentrations of EPA for 2 hours and 300 μM H_2_O_2_ or HGP for 48 hours, the supernatant was transferred into an e-tube. Isolated protein by radioimmunoprecipitation assay (RIPA) buffer was quantified by bicinchoninic Acid (BCA) kits (Pierce, CA, USA). The serum was separated by centrifugation at 300 rpm for 20 minutes. LDH was measured by LDH enzyme-linked immunosorbent assay kit (Abcam, Cambridge, UK).

### Cell cycle analysis

HUVECs were washed with phosphate-buffered saline (PBS) and slowly drop-chilled with 70% ethanol to fix the cells. After 30 minutes of incubation, cells were re-washed with PBS and re-suspended with 50 μg/mL PI (BD, CA, USA) and 50 μg/mL RNase A at room temperature. After incubation for 30 minutes in the dark, 300 μL of FACS buffer (1% FBS, 0.1% NaN_3_) was added, and analysis was performed using FACSort (BECTON DICKINSON, BD bioscience).

### Senescence-associated beta-galactosidase (SA-β-Gal) staining

After incubation under appropriate conditions in H_2_O_2_ or HGP with/without EPA, HUVECs were washed with PBS, and then SA-β-Gal working solution (pH 6.0) (Cell signaling, MA, USA) was added. After 24 hours, cells were observed under an inverted microscope. The percentages of SA-β-Gal-stained cells per total cells were compared quantitatively.

### p16 expression by immunofluorescent staining

Immunofluorescent staining was performed to determine the cellular distribution of p16. HUVECs were plated onto a cover slide. After incubation under appropriate conditions, cells were fixed and permeabilized with cytoperm/cytofix (BD, CA, USA) for 15 minutes and incubated with blocking buffer (5% serum and 0.3% triton X-100 in PBS) for 1 hour and then incubated in anti-p16 antibody (Abcam, Cambridge, UK) for 1 hour at room temperature. Then the cells were incubated in mouse-anti Alexa 488 Fluor^Ⓡ^ as a secondary antibody and 4′,6-diamidino-2-phenylindole (DAPI, 1 μg/mL) for 1 hour at room temperature. Fluorescence in situ hybridization (FISH) analysis was performed using a green fluorescence microscope, Olympus BX-51 (Olympus, Tokyo, Japan).

### Western blotting analysis

HUVECs were washed with PBS and lysed using mammalian tissue lysis/extraction reagent (Sigma, MO, USA), including protease inhibitor (Roche, IN, USA). Protein concentrations were determined by BCA protein assay kit (Pierce, CA, USA), and 1× sodium dodecyl sulfate (SDS) sample buffer (50 mM Tris, pH 6.8, 2% SDS, 10% glycerol, 50 mM dithiothreitol, and 0.01% bromophenol blue) was added. Proteins were separated in 10-15% SDS-polyacrylamide gel electrophoresis, transferred onto polyvinylidene difluoride membranes, and immunoblotted with anti-silent information regulator type 1 (Sirt1) (Abcam, Cambridge, UK), anti-phospho-p38 mitogen-activated protein kinase (MAPK) (Cell signaling, MA, USA), anti-p38 MAPK (Cell signaling, MA, USA), anti-p16 (Abcam, Cambridge, UK), anti-Bcl2 (Cell signaling, MA, USA), anti-cleaved caspase3 (Cell signaling, MA, USA), anti-phospho-AMP-activated protein kinase (AMPK) (Cell signaling, MA, USA), anti-AMPK (Cell signaling, MA, USA), and anti-glyceraldehyde 3-phosphate dehydrogenase (GAPDH) (Cell signaling, MA, USA) at 4°C overnight. Secondary antibodies (goat anti-rabbit or anti-mouse conjugated alkaline phosphatase (Bethyl, TX, USA)) were added for 1 hour at room temperature, and membranes were developed via alkaline phosphatase-conjugated development kit (Bio-rad, CA, USA). Developed protein bands were quantified by Image J program (NIH, Bethesda, MD, USA).

### Statistical analysis

Data were expressed as the mean ± standard deviation, and statistical analyses were performed using SPSS software (version 20.0; IBM Corp., NY, USA). Differences between the groups of results were determined using a one-way ANOVA, followed by a Tukey's *post hoc* test. In all analyses, P<0.05 was considered statistically significant.

## Results

### Effects of EPA treatment on cell viability and apoptosis of HUVECs under stress conditions

**Figure 1:**
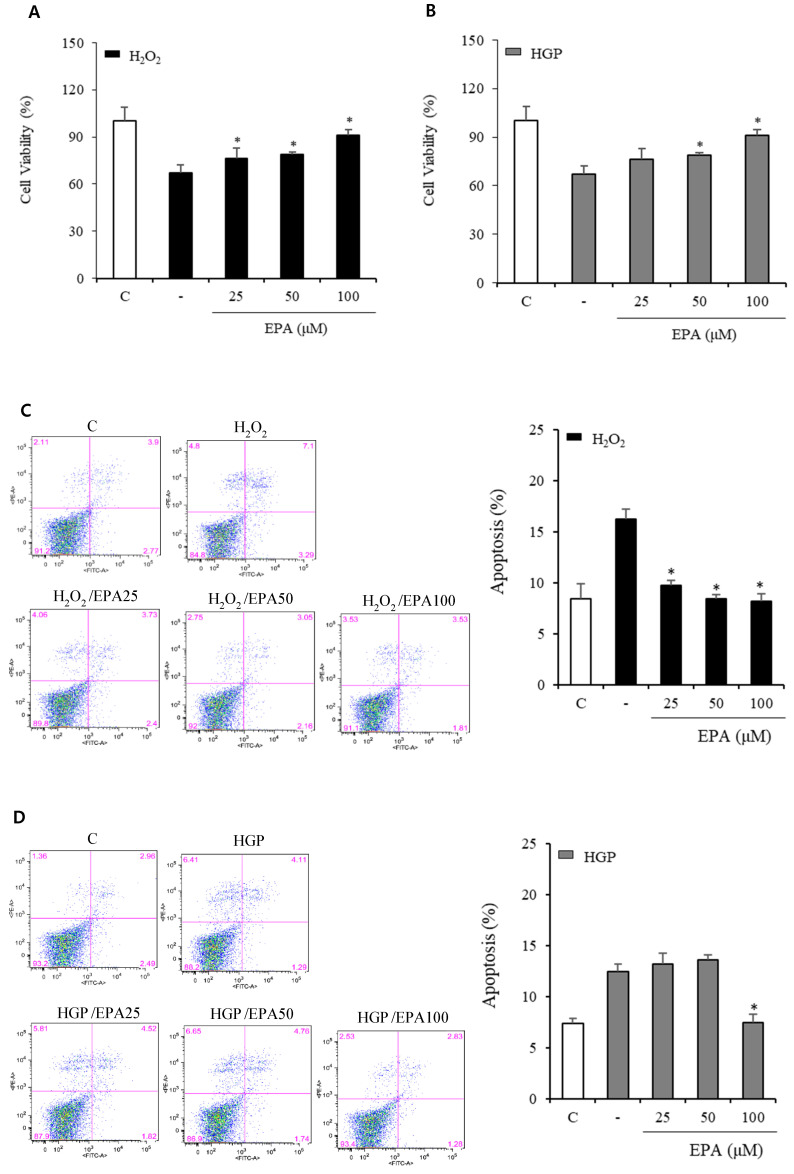
Effects of EPA on cell viability and apoptosis of HUVECs under stress conditions. (A) Cell viability measured using the CCK-8 assay was decreased by oxidative stress induced by 300 μM of H_2_O_2_, which was attenuated by treatment with 25, 50, and 100 μM EPA. (B) Cell viability measured using CCK-8 assay was decreased by HGP (25 mM glucose and 300 μM palmitate), which was attenuated by treatment with 50 and 100 μM EPA treatment. (C) Cellular apoptosis detected using Annexin V-FITC and PI double staining was increased by oxidative stress, which was attenuated by treatment with 25, 50, and 100 μM EPA. (D) Cellular apoptosis detected using Annexin V/PI staining was increased by HGP, which was attenuated by treatment with 100 μM EPA. These experiments were performed in triplicate. Values are means ± SD. C: control, EPA: eicosapentaenoic acid, CCK: cell counting kit, HGP: high glucose concentration with palmitate, PI: propidium iodide *Significantly different from the stress group, *P*<0.05.

We conducted a CCK-8 experiment to confirm whether the cell viability was decreased by H_2_O_2_ or glucolipotoxicity and if EPA had a protective effect (Figure 1A, 1B). When assessed 48 hours after EPA treatment, the protective effect of EPA on cell viability was observed at 25, 50, and 100 μM concentrations in the H_2_O_2_ group, and similar results were obtained in the control group at 100 μM of EPA. In the group exposed to HGP (25 mM glucose and 300 μM palmitate), the cell viability increased at 50 and 100 μM of EPA compared to the HGP-treated group without EPA.

At 100 μM EPA, similar results to that of the control group were obtained, but no protective effect was observed at 25 μM of EPA. After the reaction, cells were stained with annexin V/PI to observe the anti-apoptotic effect of EPA (Figure 1C, 1D). In the control group, apoptosis was approximately 8%, whereas, in the H_2_O_2_ treatment group, it was increased by approximately 16% (a two-fold increase). Apoptosis was increased by H_2_O_2_ and was decreased by EPA. The anti-apoptotic effect was confirmed at approximately 10% at 25 μM and 8% at 50 and 100 μM. The HGP-treated group exhibited 14% apoptosis, which was increased by approximately 7% compared to the control group. At 100 μM, EPA reduced the apoptosis increased by HGP as much as the control, but no difference was observed with 25 and 50 μM EPA.

**Figure 2:**
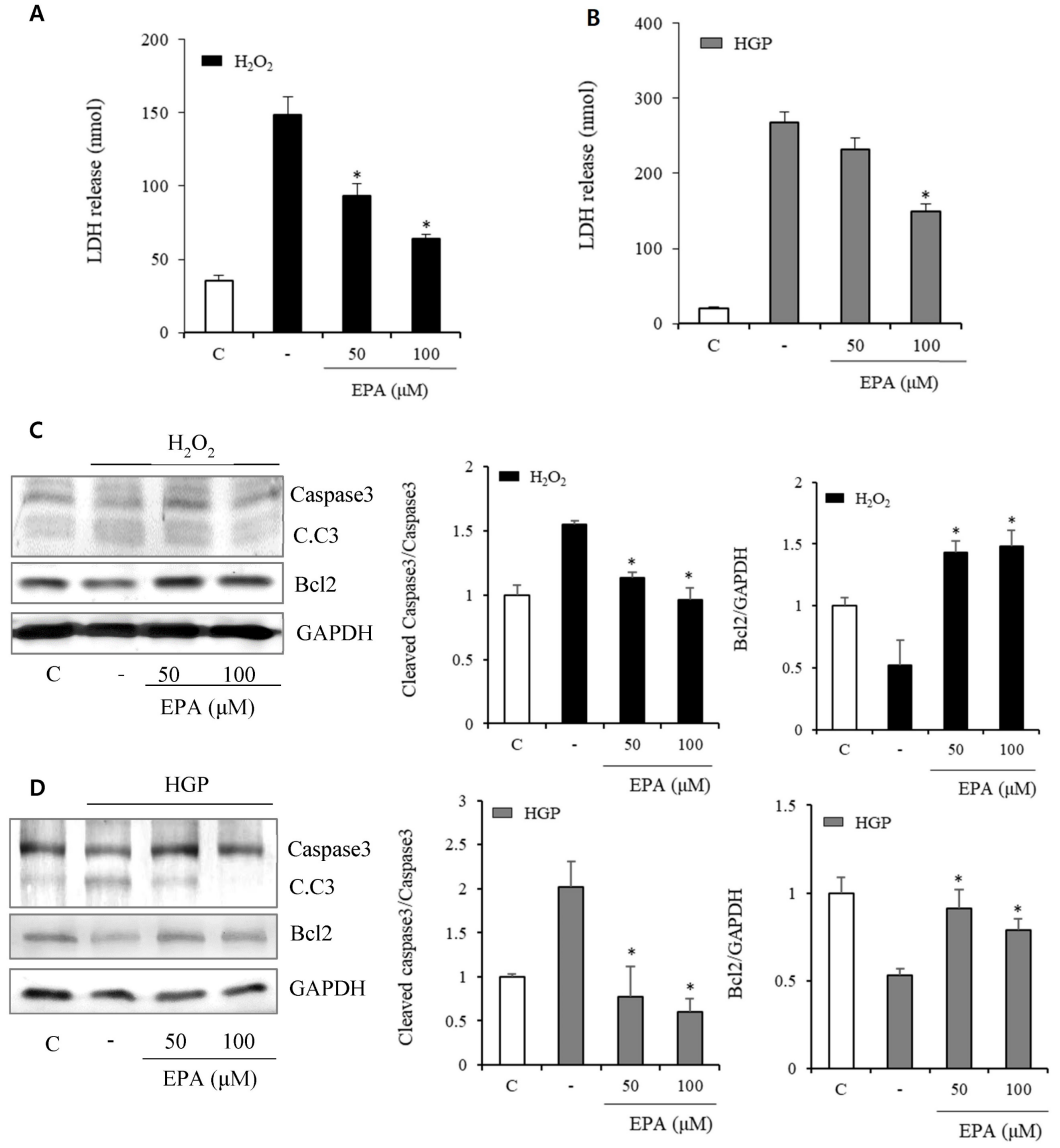
Effects of EPA on markers related to cellular death and apoptosis in HUVECs under stress conditions (A) LDH release, indicating cellular death, was significantly increased after H_2_O_2_ treatment and reduced by treatment with 50 and 100 μM EPA. (B) LDH release was significantly increased after HGP treatment and reduced by 100 μM EPA treatment. (C, D) Cleaved caspase-3 to caspase-3 ratio, a proapoptotic marker increased by H_2_O_2_ or HGP treatment, was decreased by treatment with 50 and 100 μM EPA. The Bcl2, an antiapoptotic marker decreased by H_2_O_2_ or HGP treatment, was increased by treatment with 50 and 100 μM EPA. These experiments were performed in triplicate. Values are means ± SD. C: control, C.C3: cleaved caspase-3, EPA: eicosapentaenoic acid, HGP: high glucose concentration with palmitate *Significantly different from the stress group, *P*<0.05.

In the H_2_O_2_ exposed group, LDH release indicating cellular death increased after H_2_O_2_ and was reduced by treatment with 50 and 100 μM EPA (Figure 2A). LDH markedly increased over 10 times in the HGP-treated group compared to the control group, and LDH was reduced by half after treatment with 100 μM EPA in the control group compared to the HGP-treated group. However, there was no effect at 50 μM of EPA (Figure 2B).

Anti-apoptosis-related proteins were identified (Figure 2C, 2D). Cleaved caspase3, which was increased by H_2_O_2_ treatment, was decreased by EPA. Bcl2, an anti-apoptosis protein, was also reduced by H_2_O_2_ treatment but restored by EPA. Similar results were obtained in the HGP-treated group.

**Figure 3:**
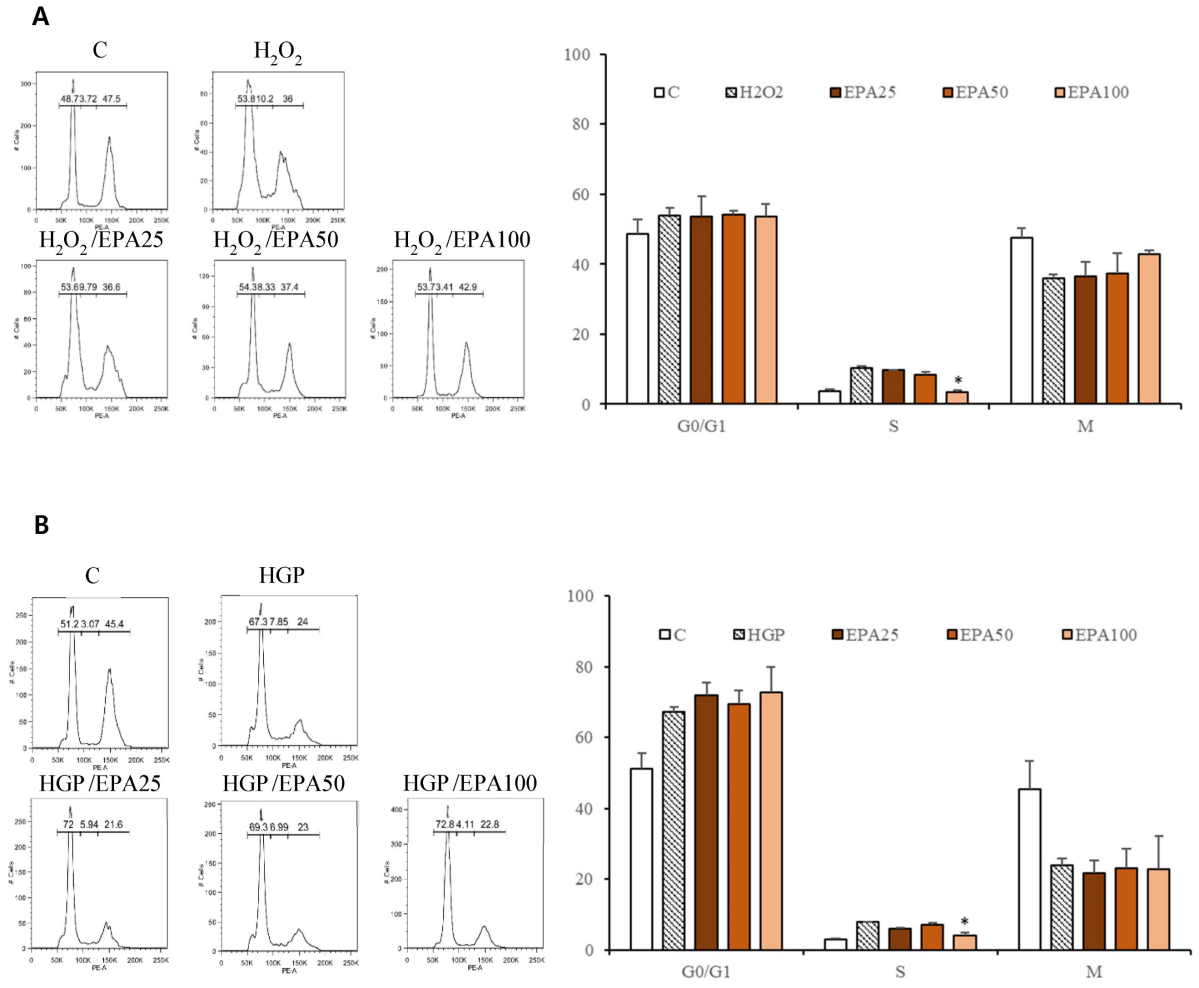
The effect of the EPA on the cell cycle in HUVECs under stress conditions. (A) HUVECs that were significantly stagnant in the s phase by H_2_O_2_ treatment (10.2%) were recovered by EPA in a dose-dependent manner (25 μM; 9.79%, 50 μM; 8.33%, 100 μM; 3.41%), although only the 100 μM concentration was significant. (B) The cells that were significantly stagnant in the s phase by HGP treatment (7.85%) were recovered by treatment with 100 μM EPA (3.41%). These experiments were performed in triplicate. Values are means ± SD. C: control, EPA: eicosapentaenoic acid, HGP: high glucose concentration with palmitate *Significantly different from the stress group, *P*<0.05.

The cell cycle was investigated (Figure 3). HUVECs that were stagnant in the s phase due to H_2_O_2_ application (10.2%) were recovered by EPA in a dose-dependent manner (25 μM; 9.79%, 50 μM; 8.33%, 100 μM; 3.41%), although only the 100 μM concentration was significant. Cells that were stagnant in the s phase by HGP (7.85%) were recovered by treatment with 100 μM EPA (4.11%).

### Effects of EPA on cellular senescence of HUVECs under stress conditions

**Figure 4:**
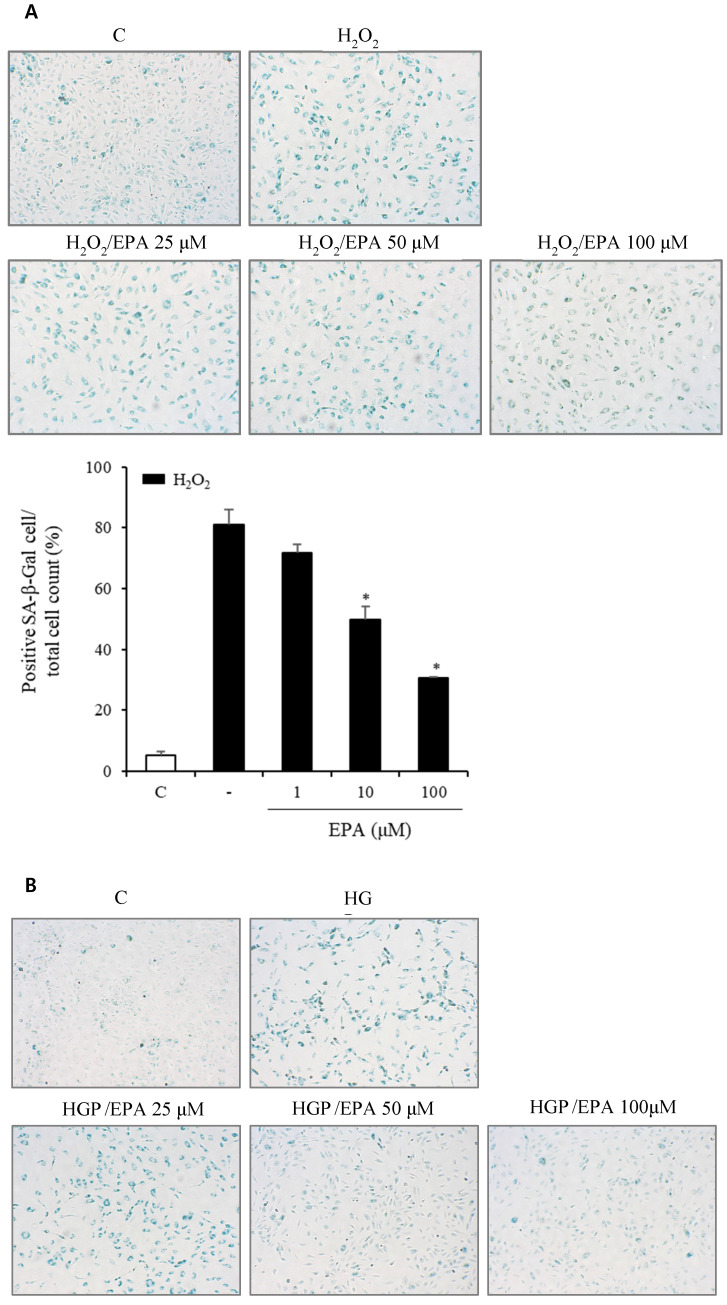

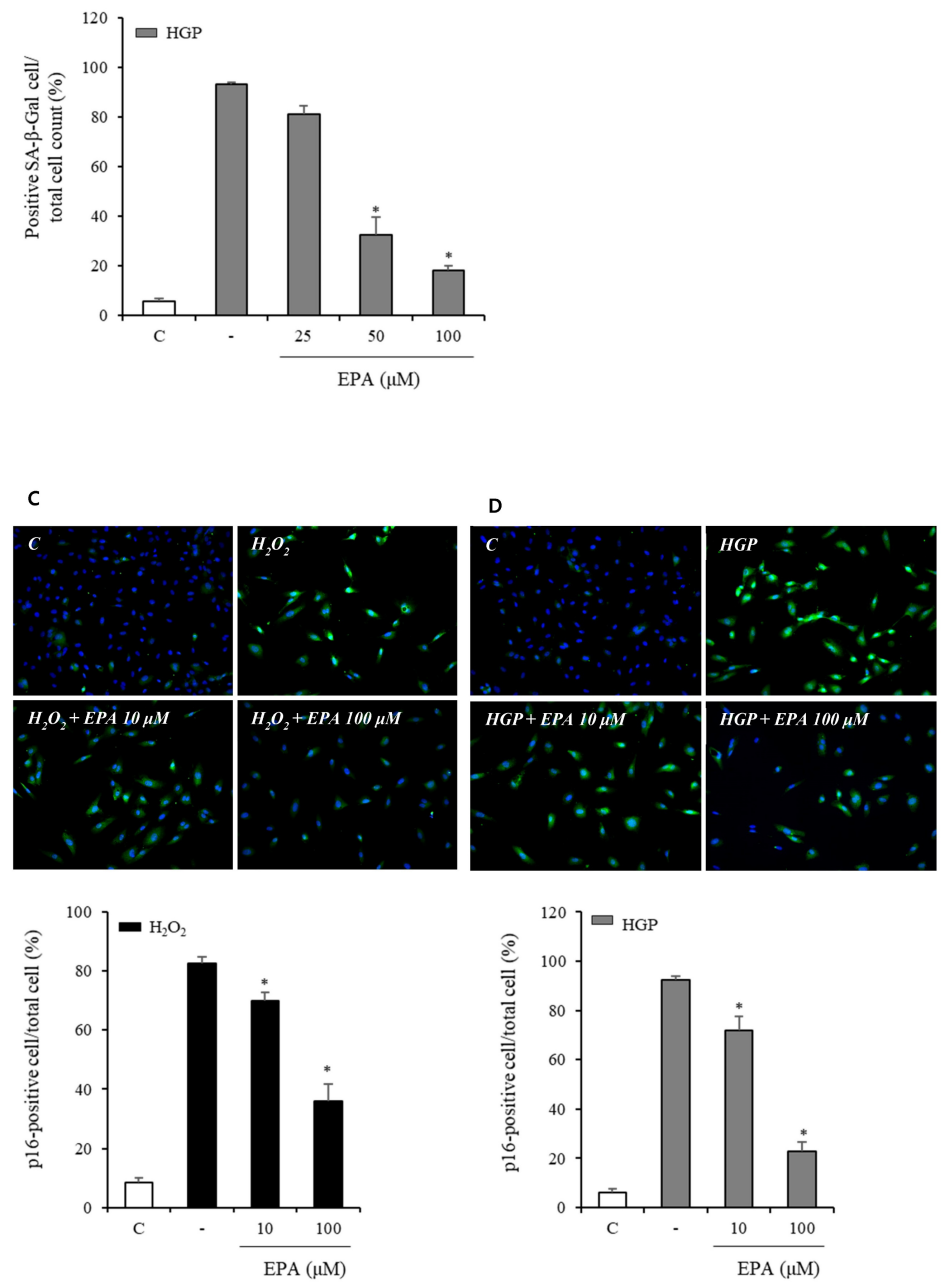
Effects of EPA on cellular senescence of HUVECs under stress conditions. (A, B) Senescent (SA-β-Gal-positive, green) cells increased after H_2_O_2_ or HGP treatment and decreased by treatment with 25, 50, and 100 μM EPA in a dose-dependent manner. (C, D) p16 protein expression (green) detected by immunofluorescent staining was increased after H_2_O_2_ or HGP treatment and decreased by treatment with 10 and 100 μM EPA, respectively. C: control, EPA: eicosapentaenoic acid, HGP: high glucose concentration with palmitate.

To observe the aging effects of H_2_O_2_ and the anti-aging effects of EPA, we conducted an SA-β-Gal stain experiment (Figure 4A, 4B). HUVECs were incubated with various concentrations of EPA for 24 hours and then treated with H_2_O_2_ for 48 hours. SA-β-Gal staining increased dramatically in the H_2_O_2_ group compared to the control group, whereas EPA inhibited aging, with a more pronounced effect at higher concentrations. The 100 μM EPA treatment group demonstrated similar aging inhibition as the control group. We also tested HGP under the same conditions as H_2_O_2_ and discovered that SA-β-Gal staining increased similarly. However, EPA effectively decreased the SA-β-Gal staining increased by HGP, and the anti-aging effect was greater at higher concentrations of EPA. We confirmed the change in the p16 protein through cell fluorescence staining, as p16 protein increases with aging in cells (Figure 4C, 4D). We observed a significant increase in the p16 protein in the H_2_O_2_ group compared to the control group.

### Anti-senescence mechanism of EPA under stress conditions

**Figure 5:**
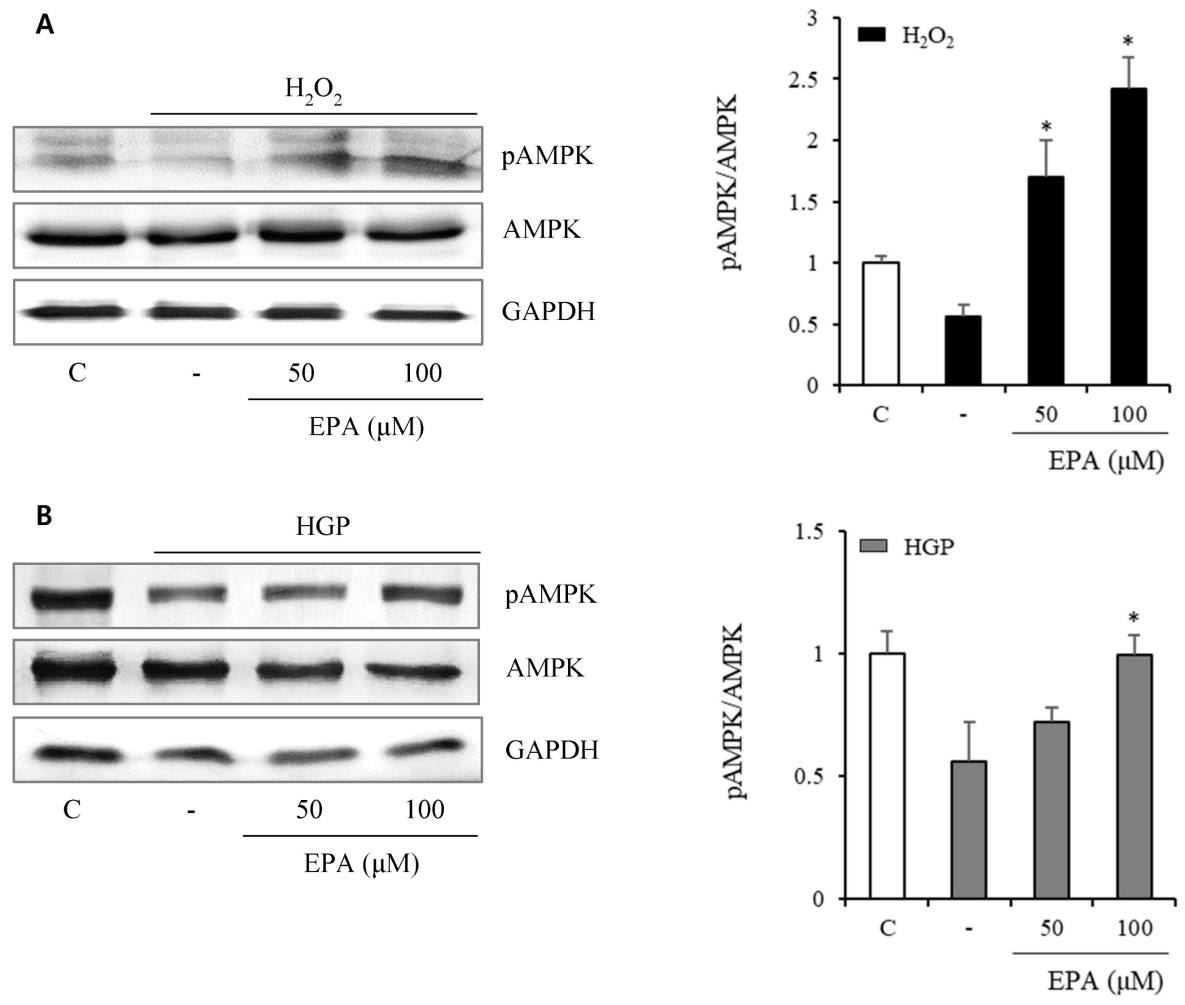
The effect of EPA on AMP‑activated protein kinase (AMPK) phosphorylation under stress conditions The AMPK phosphorylation was reduced by 50% and 25%, respectively, compared to the control under H_2_O_2_ or HGP stress conditions, which was recovered by treatment with 50 and 100 μM EPA in the H_2_O_2_ treated group, and 100 μM EPA in the HGP treated group, similar to the control group. These experiments were performed in triplicate. Values are means ± SD. C: control, EPA: eicosapentaenoic acid, HGP: high glucose concentration with palmitate *Significantly different from the stress group, *P*<0.05.

To investigate the anti-senescence mechanism, we observed changes in senescence-related proteins. AMPK phosphorylation (Figure 5) was reduced by 50% and 25% under H_2_O_2_ or HGP stress conditions, respectively, compared to the control group. However, in the H_2_O_2_-treated group, AMPK phosphorylation was recovered at 50 and 100 μM EPA. In the HGP-treated group, AMPK phosphorylation was increased at 100 μM EPA, similar to the control group.

**Figure 6:**
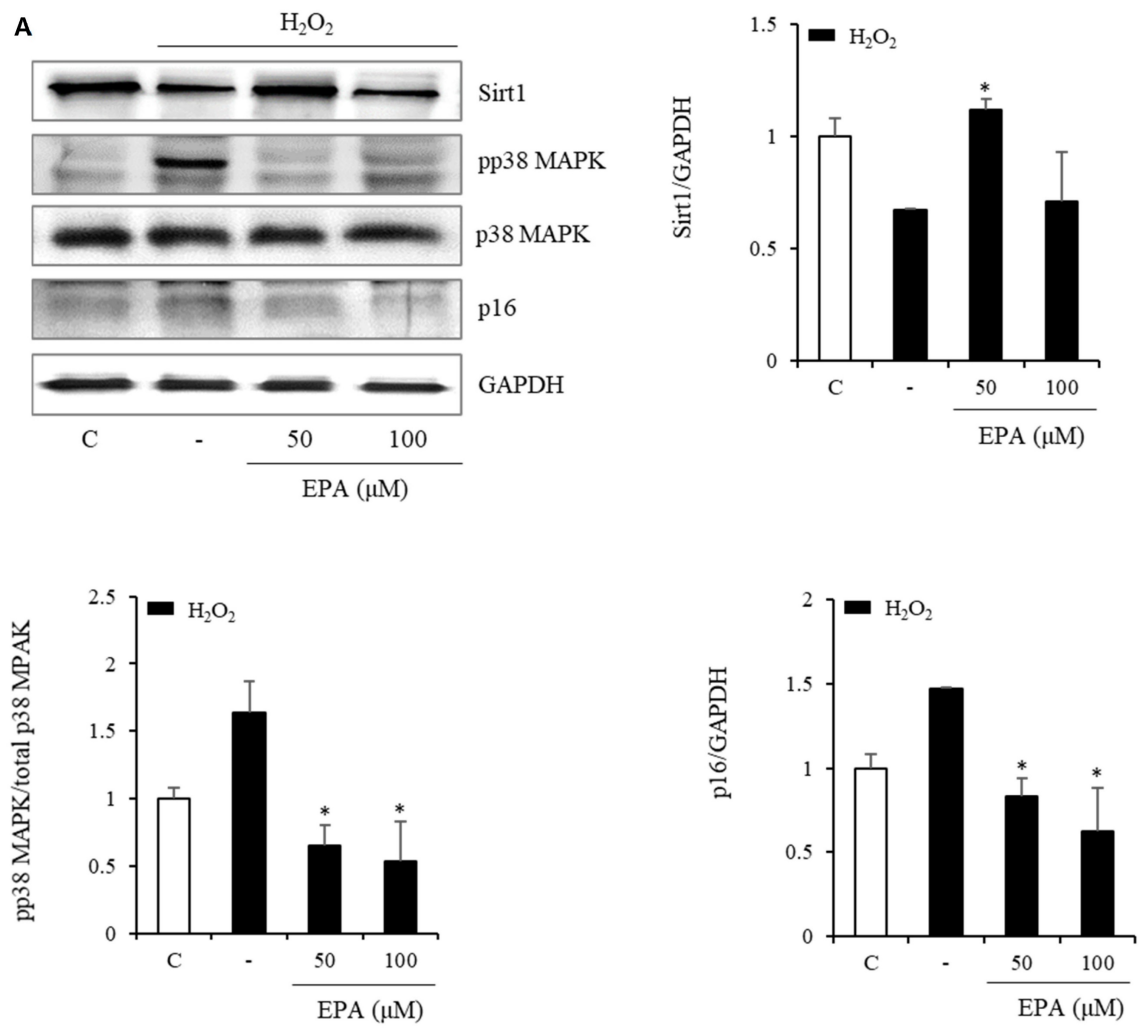

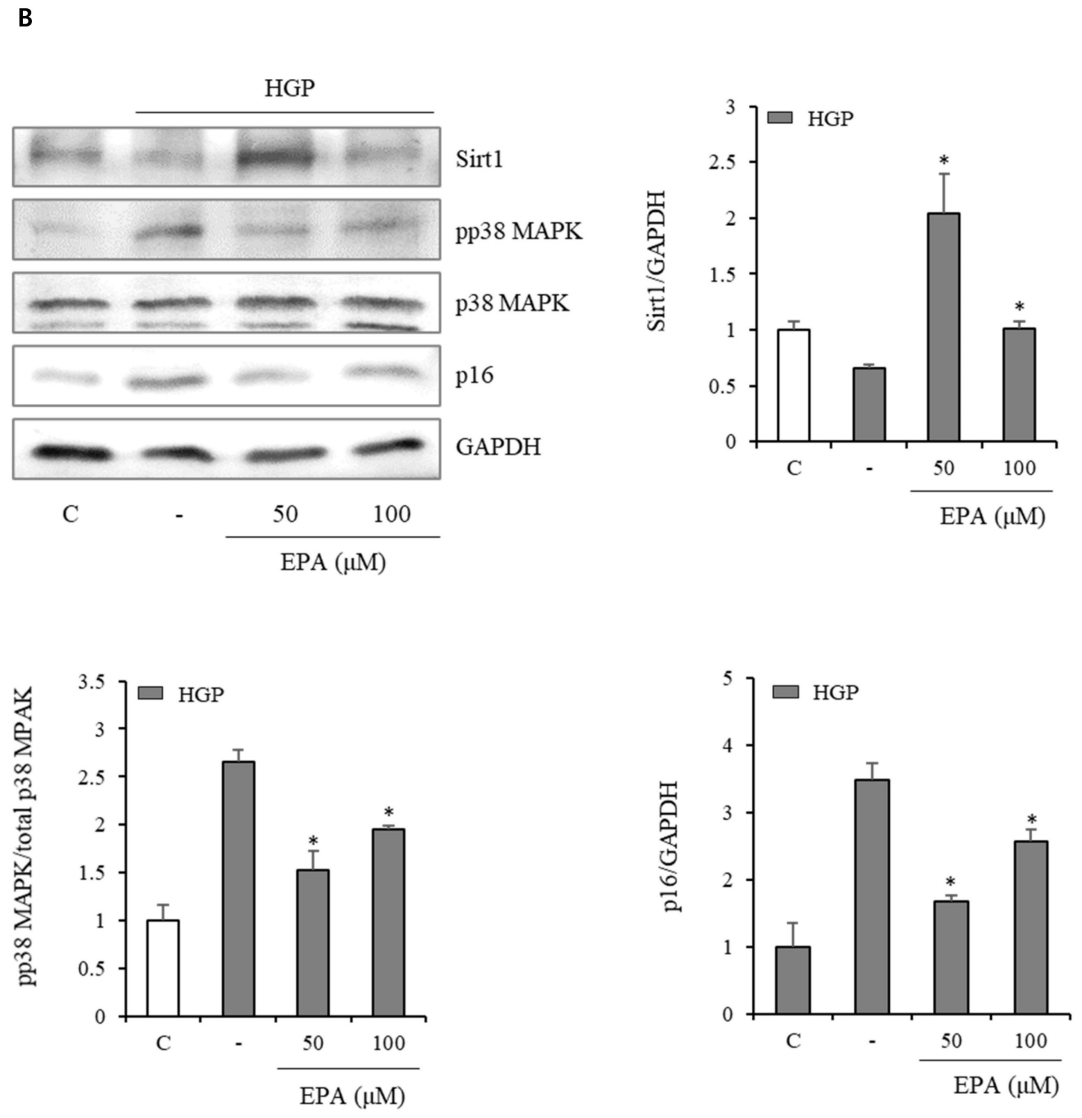
Effects of EPA on the SIRT1/p38 MAPK/p16 pathway in senescent HUVECs under stress conditions. In the H_2_O_2_ or HGP treatment group, Sirt1 was decreased, and phosphorylation of p38 MAPK and p16 protein were increased compared to the control and was recovered by EPA similar to the control group. These experiments were performed in triplicate. Values are means ± SD. C: control, EPA: eicosapentaenoic acid, HGP: high glucose concentration with palmitate *Significantly different from the stress group, *P*<0.05.

We also observed changes in the Sirt1/p38 MAPK/p16 mechanism (Figure 6). In the H_2_O_2_ treatment group, Sirt1 decreased by 50%, whereas p38 MAPK phosphorylation increased more than three-fold, and p16 protein increased two-fold. However, the EPA administration led to a recovery similar to that of the control group. This effect was also observed in the HGP treatment group.

## Discussion

Aging is a major risk factor for chronic diseases that increase with age, such as neurodegenerative disease, malignancy, metabolic disease, and cardiovascular disease. Aging and age-related diseases have common biological mechanisms, such as inflammation, the accumulation of macromolecular damage, adaptation to stressors, epigenetic changes, metabolic dysfunction, loss of proteostasis, and defective stem cell function. Major age-related diseases include type 2 diabetes, atherosclerosis, and certain cancers 17.

Atherosclerotic cardiovascular disease is one of the leading causes of mortality in patients with diabetes. Blood sugar control is important to prevent complications of diabetes. However, in large-scale clinical trials, it was difficult to confirm the short-term efficacy of strict blood sugar control in preventing cardiovascular disease complications. Rather, extremely strict glycemic control increased mortality due to the risk of hypoglycemia 18. Therefore, managing multiple risk factors, such as blood pressure and cholesterol control, is emphasized over glucose control alone 19.

Cholesterol control is important in preventing cardiovascular disease. However, even if LDL-C is sufficiently lowered using statins, there is a residual risk of cardiovascular disease of approximately 65% 20-22, and hypertriglyceridemia is a known cause 23, 24. A recent REDUCE-IT study revealed that when highly purified EPA called IPE (Vascepa^®^) was added to statins, atherosclerotic cardiovascular disease was reduced by 25% 15. Thus, various academic societies and related organizations have changed treatment guidelines 25-27. However, in the REDUCE-IT study, the reduction in triglyceride, non-HDL-C, and LDL-C due to IPE could not fully explain the cardiovascular disease prevention effect. It is believed that pleiotropic effects other than reduction may have contributed to cardiovascular disease prevention 16. These include anti-inflammatory action, antithrombotic action, cell membrane stabilizing effect, antioxidant activity, and improving the function of vascular endothelial cells and preventing arrhythmia; however, the mechanism of action has not been completely elucidated.

Cellular senescence is defined as irreversible cell cycle arrest and is activated by multiple stressors such as DNA damage, oxidative stress, and cytotoxic environments, which is stress-induced accelerated senescence (SIAS). These stimuli activate various signaling pathways through p16, p21, and p53 and activate cell cycle inhibitors. Morphologically senescent cells are usually bulky and have increased SA-β-Gal activity due to mitochondrial dysfunction and lysosomal biogenesis 28. Therefore, p16 expression and SA-β-Gal are used as biomarkers of cellular senescence. H_2_O_2-_induced oxidative stress or HGP-induced glucolipotoxicity mimicking SIAS increased the SA-β-Gal stain positivity in HUVECs, and the number of SA-β-Gal positive cells significantly decreased after EPA treatment. The expression of p16 in HUVECs was increased under oxidative stress or glucolipotoxic conditions, which decreased after EPA treatment. These results suggest that EPA protects HUVECs from SIAS.

In previous reports of normal human epidermal keratinocytes or human bone marrow-derived mesenchymal stem cells subjected to H_2_O_2_, increased ratios of cells were exposed to oxidative stress in the G0/G1 phase 29, 30. However, in this study, the percentage of cells exposed to oxidative stress in the G0/G1 phase did not increase significantly. The G0/G1 phase ratio of HUVECs exposed to glucolipotoxic conditions increased but was unaffected by EPA. The s phase was significantly increased by oxidative stress and glucolipotoxic conditions and was decreased by a high EPA concentration. There may be a difference in experimental conditions; however, additional research on cell cycle-related proteins is needed. During the s phase, the cell continuously scrutinizes its genome for abnormalities. Detection of DNA damages by oxidative and glucolipotoxic stresses induces activation of three canonical s phase "checkpoint pathways" that delay or arrest further cell cycle progression and may lead to the stagnation of cells in the s phase 31. In addition to the actions of these canonical checkpoints, recent evidence suggested that the abnormalities in histone supply and nucleosome assembly can also alter the s phase progression 32. So, the increased number of cells in the s phase after exposure to oxidative and glucolipotoxic stresses in our experiment could be interpreted as the stagnation of cells in the s phase.

On the molecular level, cellular senescence has been associated with two major intracellular signaling pathways: the p38 MAPK/p16INK4α pathway and the p53/p21 pathway 33. The protein p21, a cyclin-dependent kinase inhibitor, triggers the onset of cell cycle arrest, which can be induced by p53 34. Recently, human SIRT1, a member of the nicotinamide adenine dinucleotide-dependent deacetylase protein family, has been demonstrated to recover the senescence process by increasing cell proliferation and reducing p16INK4α expression in human diploid fibroblasts 35.

Our results revealed that Sirt1 expression was decreased in HUVECs, whereas phospho-p38 MAPK and p16 protein expressions were increased under oxidative stress or glucolipotoxic conditions, and the addition of EPA opposed the senescence marker 36.

In regulating aging and mammalian senescence, the reciprocal regulations of SIRT1 and AMPK pathways were studied 37. A decreased SIRT1 expression and activity accompany the progression from pre-senescence to irreversible senescence and an augmented AMPK function. From our study, it is difficult to interpret the relationship between AMPK phosphorylation and SIRT1 reciprocally, similar to previous studies. Therefore, additional research is needed.

This study had several limitations. First, there are apparent differences between senescence and SIAS. p16 protein and SA-β-Gal staining are considered biomarkers of senescence; however, they could be elevated under other conditions, such as inflammation. Second, we used only HUVECs in our study. However, atherosclerotic plaque builds up in arteries and is produced by the interaction of vascular endothelial cells, vascular smooth muscle cells, and inflammatory molecules 38. Third, we did not experiment with EPA-induced changes under non stressful conditions to compare oxidative stress or glucolipotoxic conditions. And EPA is a potent inducer of NO, and this may contribute to the findings of EPA's cell protection efficacies in our experiments. Additional experiments are needed.

## Conclusions

In conclusion, the senescence-related process is partially involved in the development of atherosclerosis and diabetic complications. EPA acts by ameliorating SIAS of HUVECs in oxidative stress or glucolipotoxic environment. These findings suggest that the antisenescence mechanism could be one of the pleotrophic effects of EPA.

## Abbreviations

SASP: senescent-associated secretory phenotype
REDUCE-IT: Reduction of Cardiovascular Events with Icosapent Ethyl-Intervention Trial
IPE: icosapent ethyl
ROS: reactive oxygen species
EPA: eicosapentaenoic acid
LDL-C: low-density lipoprotein cholesterol
non-HDL-C: non-high-density lipoprotein cholesterol
HUVECs: human umbilical vein endothelial cells
HGP: high glucose concentration with palmitate
PI: propidium iodide
LDH: lactate dehydrogenase
PBS: phosphate-buffered saline
SA-β-Gal: senescence-associated beta-galactosidase
SDS: sodium dodecyl sulfate
MAPK: mitogen-activated protein kinase
SIAS: stress-induced accelerated senescence
